# Postoperative lead migration in deep brain stimulation surgery: Incidence, risk factors, and clinical impact

**DOI:** 10.1371/journal.pone.0183711

**Published:** 2017-09-13

**Authors:** Takashi Morishita, Justin D. Hilliard, Michael S. Okun, Dan Neal, Kelsey A. Nestor, David Peace, Alden A. Hozouri, Mark R. Davidson, Francis J. Bova, Justin M. Sporrer, Genko Oyama, Kelly D. Foote

**Affiliations:** 1 Department of Neurosurgery, Fukuoka University, Fukuoka, Japan; 2 Department of Neurosurgery, University of Florida, Center for Movement Disorders and Neurorestoration, Gainesville, Florida, United States of America; 3 Department of Neurology, University of Florida, Center for Movement Disorders and Neurorestoration, Gainesville, Florida, United States of America; 4 Department of Materials Science and Engineering, University of Florida, Gainesville, Florida, United States of America; 5 Department of Neurology, Juntendo University, Tokyo, Japan; Oslo Universitetssykehus, NORWAY

## Abstract

**Introduction:**

Deep brain stimulation (DBS) is an effective treatment for multiple movement disorders and shows substantial promise for the treatment of some neuropsychiatric and other disorders of brain neurocircuitry. Optimal neuroanatomical lead position is a critical determinant of clinical outcomes in DBS surgery. Lead migration, defined as an unintended post-operative displacement of the DBS lead, has been previously reported. Despite several reports, however, there have been no systematic investigations of this issue. This study aimed to: 1) quantify the incidence of lead migration in a large series of DBS patients, 2) identify potential risk factors contributing to DBS lead migration, and 3) investigate the practical importance of this complication by correlating its occurrence with clinical outcomes.

**Methods:**

A database of all DBS procedures performed at UF was queried for patients who had undergone multiple post-operative DBS lead localization imaging studies separated by at least two months. Bilateral DBS implantation has commonly been performed as a staged procedure at UF, with an interval of six or more months between sides. To localize the position of each DBS lead, a head CT is acquired ~4 weeks after lead implantation and fused to the pre-operative targeting MRI. The fused targeting images (MR + stereotactic CT) acquired in preparation for the delayed second side lead implantation provide an opportunity to repeat the localization of the first implanted lead. This paradigm offers an ideal patient population for the study of delayed DBS lead migration because it provides a large cohort of patients with localization of the same implanted DBS lead at two time points. The position of the tip of each implanted DBS lead was measured on both the initial post-operative lead localization CT and the delayed CT. Lead tip displacement, intracranial lead length, and ventricular indices were collected and analyzed. Clinical outcomes were characterized with validated rating scales for all cases, and a comparison was made between outcomes of cases with lead migration versus those where migration of the lead did not occur.

**Results:**

Data from 138 leads in 132 patients with initial and delayed lead localization CT scans were analyzed. The mean distance between initial and delayed DBS lead tip position was 2.2 mm and the mean change in intracranial lead length was 0.45 mm. Significant delayed migration (>3 mm) was observed in 17 leads in 16 patients (12.3% of leads, 12.1% of patients). Factors associated with lead migration were: technical error, repetitive dystonic head movement, and twiddler’s syndrome. Outcomes were worse in dystonia patients with lead migration (p = 0.035). In the PD group, worse clinical outcomes trended in cases with lead migration.

**Conclusions:**

Over 10% of DBS leads in this large single center cohort were displaced by greater than 3 mm on delayed measurement, adversely affecting outcomes. Multiple risk factors emerged, including technical error during implantation of the DBS pulse generator and failure of lead fixation at the burr hole site. We hypothesize that a change in surgical technique and a more effective lead fixation device might mitigate this problem.

## Introduction

Deep brain stimulation (DBS) has proven to be a highly effective therapy for select patients with movement and neuropsychiatric disorders [1–4].The neural structures targeted for DBS lead implantation are on the order of millimeters in dimension and are surrounded by structures that, if stimulated, may cause untoward side effects. Optimal lead placement is a critical factor in the success or failure of DBS therapy [5–7]. Suboptimal neuroanatomical lead position adversely affects clinical outcomes, and in select cases, lead repositioning has improved DBS outcomes significantly [7].

Delayed migration of a DBS lead is a known hardware related complication that can adversely affect clinical outcome [5]. Multiple authors have reported on small series of lead migrations and have postulated possible mechanisms underpinning this complication [8, 9]. The incidence of lead migration varies among reported series [10–20]. The majority of studies have reported DBS lead migration incidence based on malpositioned leads discovered on delayed imaging studies performed to evaluate ineffective DBS [8, 10, 11, 14, 16, 19], but none have employed detailed measurements on serial high resolution images, or correlated validated clinical outcome measures to determine the clinical impact of DBS lead migration. This study aimed to quantify the incidence of lead migration in a large cohort of DBS patients and to identify potential risk factors contributing to delayed DBS lead migration.

The UF DBS clinical cohort provides a unique opportunity to examine delayed lead migrations, since patients in our practice commonly undergo staged bilateral operations, typically with six or more months lapsing between the two lead implantation procedures. Our standard protocol includes high-resolution pre-operative targeting imaging and high-resolution lead localization imaging one month after every DBS lead implantation. The delayed staging of bilateral lead implantations requires repeat targeting imaging prior to implantation of the second lead, which provides an opportunity to precisely localize the initially implanted DBS lead at two points separated in time by a minimum of 2 months (typically > 6 months) for comparison. We also aimed to measure the clinical impact, if any, of delayed lead migration through correlation with validated outcome measures.

## Methods

### UF staged DBS implantation and lead localization protocols

**Fig 1:**
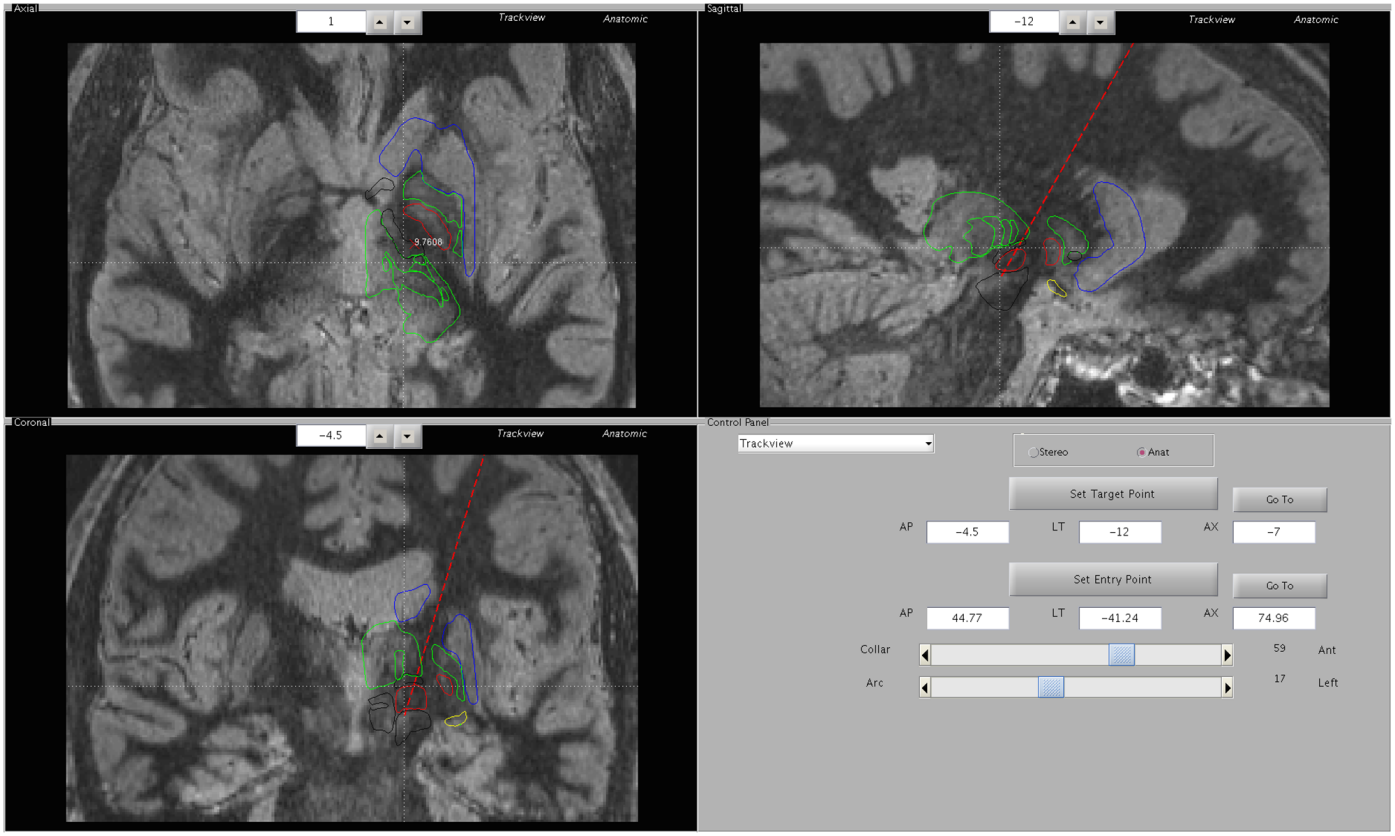
Graphical depiction of the measured neuroanatomical position of a DBS lead. **The precise position of the implanted DBS lead is measured on a high-resolution CT scan acquired a few weeks after implantation and fused to the pre-operative targeting images** (FGATIR MRI sequence with patient-specific deformed 3D atlas overlay).

DBS implantation is commonly performed as a staged procedure at our institution as follows: Stage 1) unilateral DBS lead implantation under local anesthesia with hospital discharge on post-operative day one, Stage 2) pulse generator implantation and activation approximately one month later as an outpatient procedure. DBS targeting is performed with 3T MRI (Gadolinium enhanced MPRAGE and FGATIR[21] sequences) with a patient-specific, 3-dimensional deformable brain atlas overlay[22] fused to high-resolution stereotactic CT images. For quality control, and to assist with DBS programming, the precise neuroanatomical location of each implanted DBS lead is measured and its position is depicted graphically relative to surrounding neuroanatomical structures. Immediate post-lead implantation imaging is not performed unless clinically indicated, but a high-resolution head CT is routinely acquired and fused to the pre-operative targeting MRI as part of the pre-operative evaluation on the day preceding pulse generator implantation. The one-month delay prior to acquisition of the post-operative lead localization CT allows sufficient time for any brain shift from the lead implantation procedure to resolve and optimizes accuracy of image fusion and lead localization. A 3-dimensional graphical depiction of each implanted DBS lead is generated on the targeting MRI with a carefully matched, patient-specific atlas overlay (Fig 1). Given the known variation among patients in the Cartesian coordinates of clinically important neuroanatomical structures relative to the mid-commissural point, and the high-quality imaging and 3-dimensional targeting software now available, this type of direct, patient-specific, neuroanatomical lead localization is more meaningful and far more clinically useful than historically common methods of indirect targeting and DBS lead localization with simple Cartesian coordinates.

At UF, unilateral DBS therapy is typically optimized for 6 months, at which time a systematic multidisciplinary re-evaluation is performed for each patient and standardized outcomes data are entered into the UF “INFORM” movement disorders research database. This study was approved by University of Florida Institutional Review Board—approval: #274–2012. All adult participants provided written informed consent to be included in the database from which the data in this study were retrospectively drawn. The results of this 6-month post-op evaluation are compared to those of the similar pre-operative evaluation to carefully assess both the positive and the negative impact of DBS therapy on a broad array of clinical outcome measures for each patient. These detailed outcomes assessments are useful for quality control and for optimization of DBS therapy for individual patients. More importantly, however, careful studies reviewing various aspects of this extensive DBS-related data from over 1400 lead implantation procedures have led to a better understanding of both positive and negative effects of DBS, and to many modifications and refinements of our DBS methodology that have reduced adverse effects and improved clinical benefit for DBS patients over the past 15 years. After the 6-month post-operative evaluation, those unilaterally implanted patients who opt to proceed with addition of a contralateral DBS lead then repeat this protocol for the second side.

### Study protocol

To address questions regarding delayed DBS lead migration, we performed a retrospective review of the UF INFORM database to identify all patients who had undergone at least two separate high-resolution CT imaging studies after DBS lead implantation. Some of the patients identified underwent delayed lead localization imaging to evaluate suboptimal clinical results, but the majority were patients who had repeat imaging for targeting of a delayed staged contralateral DBS lead implantation. Among the identified patients, two cases with poor image quality were excluded due to inability to precisely measure lead location. One pediatric case was also excluded from this analysis. Skull growth was the documented reason for lead migration in three pediatric cases, and this unusual occurrence would not be expected to affect the general population. Also excluded were four cases in which a second procedure was performed to re-implant a DBS lead in the same target following removal of a DBS lead due to infection or lead fracture. The details of all cases have been summarized in Table 1.

**Table 1:** Patient characteristics.

	Overall (n = 138)	Migration (n = 17, 12.3%)	Non-migration (n = 121, 87.7%)	p-value†
**Diagnosis**				
Dystonia-primary	12 (8.7%)	0 (0%)	12 (9.9%)	0.040* for test of all 4 categories; 0.754* if we combine dystonia categories
Dystonia-secondary	6 (4.3%)	3 (17.6%)	3 (2.5%)	
ET	16 (11.6%)	2 (11.8%)	14 (11.6%)	
PD	104 (75.4%)	13 (70.6%)	91 (76.0%)	
**Age**				
mean (SD); median (range)	57.9 (12.8); 61 (15, 81)	54.8 (19.6); 63 (15, 79)	58.3 (11.6); 61 (19, 81)	0.917**
**Gender**				
Female	45 (32.6%)	8 (47.1%)	37 (30.6%)	0.180*
Male	93 (67.4%)	9 (52.9%)	83 (69.4%)	
**Target**				
GPI	39 (28.3%)	6 (35.3%)	33 (27.3%)	0.802*
STN	83 (60.1%)	9 (52.9%)	74 (61.2%)	
Vim	16 (11.6%)	2 (11.8%)	14 (11.6%)	
**Side**				
L	85 (61.6%)	12 (70.6%)	73 (60.03	0.595*
R	53 (38.4%)	5 (29.4%)	48 (39.7%)	
**Days between two CT scans**				
mean (SD); median (range)	412 (306); 266 (14, 1659)	388 (251); 259 (56, 784)	415 (314); 280 (14, 1659)	0.841**
**Year (row percentages)**				
2002	1 (0.7%)	0 (0%)	1 (100%)	0.0004* for test of association between migration and ‘02-’07 vs. ‘08-‘11
2003	12 (8.7%)	0 (0%)	12 (100%)	
2004	8 (5.8%)	0 (0%)	8 (100%)	
2005	13 (9.4%)	1 (7.7%)	12 (92.3%)	
2006	23 (16.7%)	1 (4.3%)	22 (95.7%)	
2007	23 (16.7%)	1 (4.3%)	22 (95.7%)	
2008	16 (11.6%)	3 (18.8%)	13 (81.2%)	
2009	12 (8.7%)	6 (50.0%)	6 (50.0%)	
2010	20 (14.5%)	5 (25.0%)	15 (75.0%)	
2011	10 (7.2%)	0 (0%)	10 (100%)	
**Ventricular index**				
mean (SD); median (range)	0.330 (.219); 0.308 (.142, 2.62)	0.325 (.051); 0.325 (.234, .397)	0.330 (.233); 0.307 (.142, 2.62)	0.123
**Evans index**				
mean (SD); median (range)	0.274 (.086); 0.270 (.123, 1.12)	0.285 (.049); 0.273 (.207, .386)	0.273 (.090); 0.270 (.123, 1.12)	0.256
**Cella media index**				
mean (SD); median (range)	0.260 (.265); 0.199 (.125, 2.29)	0.242 (.182); 0.199 (.141, .933)	0.263 (.275); 0.200 (.125, 2.29)	0.819

^**†**^p-values are the results of

(*) Fisher’s exact test or

(**) Mann-Whitney U-Test

ET = essential tremor, GPI = globus pallidus interna, PD = Parkinson’s disease

The AC-PC coordinates of the tip of each DBS lead (C) and the intracranial lead length (L) were carefully measured on both the initial post-op lead localization imaging study, and on the delayed study. Any change in lead tip position (ΔC) or intracranial lead length (ΔL) between the two imaging studies was documented for each case. One previous study reported that postoperative curving of the intracranial DBS lead might result in dorsal lead migration [23], which seemed a reasonable mechanistic hypothesis. The curvilinear intracranial lead length (L) was measured to differentiate between lead migration due to intracranial lead curvature (in which cases ΔL would be zero) and lead migration due to failure at the skull fixation point (in which cases ΔL would approximate the lead migration ΔC). A statistical analysis was performed to estimate the measurement error. Taking this estimation into account (details are described in the “Results” section) and considering the 3-mm distance between adjacent contacts on the Medtronic 3387 lead that we typically use, a significant lead migration was defined as a change greater than 3 mm in lead position (ΔC) or intracranial lead length (ΔL).

A chart review was performed to obtain a detailed history for each identified case of significant DBS lead migration. A statistical analysis was performed to identify risk factors for lead migration. The variables analyzed included diagnosis, age, gender, DBS target, DBS side, and days elapsed between the two DBS lead localization CT scans. Also included were several ventricular indices including the Evans’ index, the ventricular index, and the cella-media index (all measures of brain atrophy). These were included to test the hypothesis that increased brain atrophy might be a risk factor for post-operative intracranial lead curving postulated as one potential mechanism for dorsal migration of DBS leads [23].

Clinical outcomes of patients in the entire study cohort with or without significant lead migration were analyzed. We used the Unified Dystonia Rating Scale (UDRS) total scores for dystonia cases, the tremor rating scale (TRS) motor scores for essential tremor (ET) cases, and the Unified Parkinson’s Disease Rating Scale (UPDRS) motor scores for objective evaluation of clinical outcomes. The percent change in validated outcome scale scores between the pre-operative baseline score and the score measured at the last clinical follow-up prior to delayed lead localization imaging were calculated and compared between the groups with and without significant delayed migration of their DBS leads. DBS programming data for each case was also summarized and evaluated.

### Surgical procedures

We typically perform unilateral DBS lead implantations for most PD and ET patients, commonly with the addition of a contralateral DBS system as a staged procedure several months later as described above. On the other hand, we have employed bilateral simultaneous or rapidly staged (approximately one month interval between the two lead implantation procedures) DBS lead implantation techniques for patients with dystonia, obsessive-compulsive disorder (Goodman *et al*., 2010), Tourette’s syndrome [3, 24] and constant current PD study patients [2], along with some younger, low risk PD patients with bilaterally problematic symptoms for whom there was some compelling reason to expedite bilateral lead implantation (e.g. long distance travel to the center for surgery or urgent need to return to work). Since patients who underwent bilateral simultaneous or rapidly staged bilateral lead implantations have generally undergone only a single post-operative lead localization imaging study, and were therefore not candidates for the present study, we describe here our standardized surgical protocol for delayed staged bilateral DBS patients:

#### “DBS Part 1”: Lead implantation

**Targeting**: A high-resolution (1x1x1 mm voxel) 3 Tesla MRI targeting study (including a Gadolinium enhanced MPRAGE sequence to clearly delineate cortical and ventricular surfaces and vascular anatomy, and an FGATIR sequence to enable clear identification of pertinent deep brain structures) is typically acquired one day prior to DBS lead implantation. On the day of the lead implantation procedure, a Cosman-Roberts-Wells (CRW) frame is attached to the head using local anesthesia and a stereotactic, *high-resolution (1 mm slice thickness) CT* scan is acquired. Using specialized stereotactic targeting and brain mapping software developed at the University of Florida, the following steps are performed:

- The MR and CT images are fused,
- A Cartesian coordinate system centered on the mid-commissural point is developed by carefully identifying the ventricular surfaces of the anterior and posterior commissures and the median plane, and the brain is displayed in orthogonal anatomic space based on this AC-PC coordinate system,
- A deformable 3-dimensional digital brain atlas is rotated, translated, and scaled along each of the three axes to achieve the highest level of conformity possible between the 3-dimensional atlas overlay and the individual patient’s brain,
- The stereotactic target and trans-frontal trajectory are planned using *direct targeting*, paying careful attention to both the ultimate neuroanatomical position of the planned DBS electrode array (facilitated by the graphical 3D representation of the planned lead position relative to pertinent neuroanatomic structures in MRI space enhanced by the conformal 3D atlas) and the avoidance of cortical and periventricular veins, ventricles and sulci.

**Burr hole**: After completion of DBS targeting, the stereotactic coordinates are independently set on both the CRW arc and a *phantom patient*. The arc is initially mated to the phantom to confirm the accuracy of the stereotactic system, then transferred to the patient’s head ring and secured. The entry point is stereotactically marked on the scalp, the site is infiltrated with local anesthetic, and a linear parasagittal incision is made. A standard 14 mm burr hole is placed at the stereotactically defined entry site and (since 2011) a 2-mm deep depression is drilled in the outer surface of the skull surrounding the burr hole conforming to the shape of the Navigus Stimloc^™^ Burr Hole Cover system (Medtronic, Minneapolis, MN, USA) in order to countersink the Stimloc^™^ cap to make it flush with the outer table of the skull. This countersinking technique results in markedly improved cosmesis and increased patient satisfaction. It also diminishes the risk of delayed scalp erosion over the site of prominent implanted DBS hardware. After completion of the drilling, the bone is waxed and the base ring of the Stimloc system is secured into its countersunk position using the two small stainless steel screws provided. The dura is opened and coagulated with bipolar cautery. The leptomeninges are coagulated and opened to avoid excessive downward displacement of the surface of the brain during brain penetration, which can increase the risk of subdural hematoma. Measures taken to minimize intra-operative CSF loss and brain shift include positioning the patient in recumbent position with the burr hole site near the highest point in the operative field, and careful placement of Saline soaked Gelfoam^®^ (Pfizer, New York City, NY, USA) around the penetrating guide cannulae.

**Microelectrode recording (MER)** is performed to develop a limited physiologic map of the region of the selected target. The mapping software displays the three-dimensional map graphically on the MRI with conformal atlas in real time and the MER findings are used to make small adjustments to optimize the final DBS target selection.

After completion of MER, a Medtronic model 3387 DBS lead is implanted at the selected target, and **intra-operative macrostimulation** is performed via the implanted DBS lead to evaluate threshold levels for stimulation-induced side effects as well as therapeutic effects of stimulation at each contact. If the side effect profile is appropriate, the DBS lead is fixed to the skull using the remainder of the Stimloc^™^ cap assembly and intra-operative fluoroscopy is used to ensure that the newly implanted lead is not displaced during this process. A dummy connector is attached to the distal end of the DBS lead, the dummy connector and distal lead are implanted under the ipsilateral parietal scalp, and the frontal incision is closed. After lead implantation, patients are observed in the hospital overnight and typically discharged the following day.

#### “DBS Part 2”: Lead localization, pulse generator implantation and initial activation

Lead localization, pulse generator implantation and initial DBS activation are typically performed as an outpatient procedure approximately one month after DBS lead implantation.

For quality control, and to assist with DBS programming, the precise neuroanatomical location of each implanted DBS lead is measured and depicted graphically on the targeting MRI with a carefully matched, patient-specific 3D atlas overlay to illustrate the position of the implanted electrode array relative to pertinent neuroanatomical structures. A **high-resolution post-operative head CT** is acquired and fused to the pre-operative targeting MRI as part of the pre-operative evaluation on the day preceding pulse generator implantation. Thin slice CT provides a more spatially accurate measurement of DBS lead location than MRI, and the one month delay prior to acquisition of the post-operative lead localization CT allows sufficient time for any brain shift from the lead implantation procedure to resolve, thus optimizing the accuracy of image fusion and lead localization. The 3-dimensional graphical depiction of the precise neuroanatomical position of each implanted DBS lead enables the DBS programmer to more effectively predict at which contact stimulation is likely to be most clinically effective.

During the DBS Part 2 operative procedure, a pocket is developed in the subclavicular anterior chest wall deep to the pectoralis fascia (or deep to the muscle in very thin patients) to accommodate the implantable pulse generator (IPG). An incision is made in the parietal scalp overlying the palpable dummy connector marking the end of the previously implanted DBS lead. **The dummy connector is delivered from this incision along with sufficient length of the DBS lead to connect the tunneled extension cable**. The technique employed during this step of the procedure has been modified as a result of findings of this study (see explanation in the Discussion below under **“An Important Technical Error.”**) A third small (2 cm) vertical incision is made overlying the angle of the mastoid bone 3 cm posterior to the insertion of the pinna to facilitate tunneling. The tunneling tool is bent and passed from the occipital to the subclavicular incision in the plane immediately deep to the fascia overlying the posterior cervical muscles that insert on the mastoid bone. Care is taken when passing the tunneling tool from the parietal to the occipital incision to ensure that the galea is not breached, so the galea can be closed over the tunneled cable. The previously implanted lead is connected to the tunneled cable, the cable is connected to the IPG, impedance testing confirms the electronic integrity of all circuits, and the incisions are closed.

Following pulse generator implantation, the DBS system is activated and initial programming is performed when the patient has awakened sufficiently from general anesthesia. The first formal programming session is typically scheduled in the DBS programming clinic within two weeks of initial DBS activation. Formal programming sessions are scheduled monthly thereafter for up to six months as necessary to optimize DBS parameters and medication adjustments. **Formal post-operative evaluations, including standardized outcomes scales, are performed at six months post-op**. Those patients who opt to proceed with contralateral lead implantation then repeat the same implantation protocol, including repeat acquisition of high-resolution CT and MR targeting images for the contralateral lead implantation, which provides the opportunity to repeat precise localization of the initial DBS lead to determine whether any delayed displacement has occurred.

### Measurement of lead location, intracranial lead lengths, and ventricular indices

The coordinates of the tip of each DBS lead (C) relative to the mid commissural point were carefully measured on both the initial post-operative lead localization images and the delayed lead localization images for each case. The vector distance between these two points (ΔC) was calculated to determine the amount of delayed displacement of the DBS lead.

**Fig 2:**
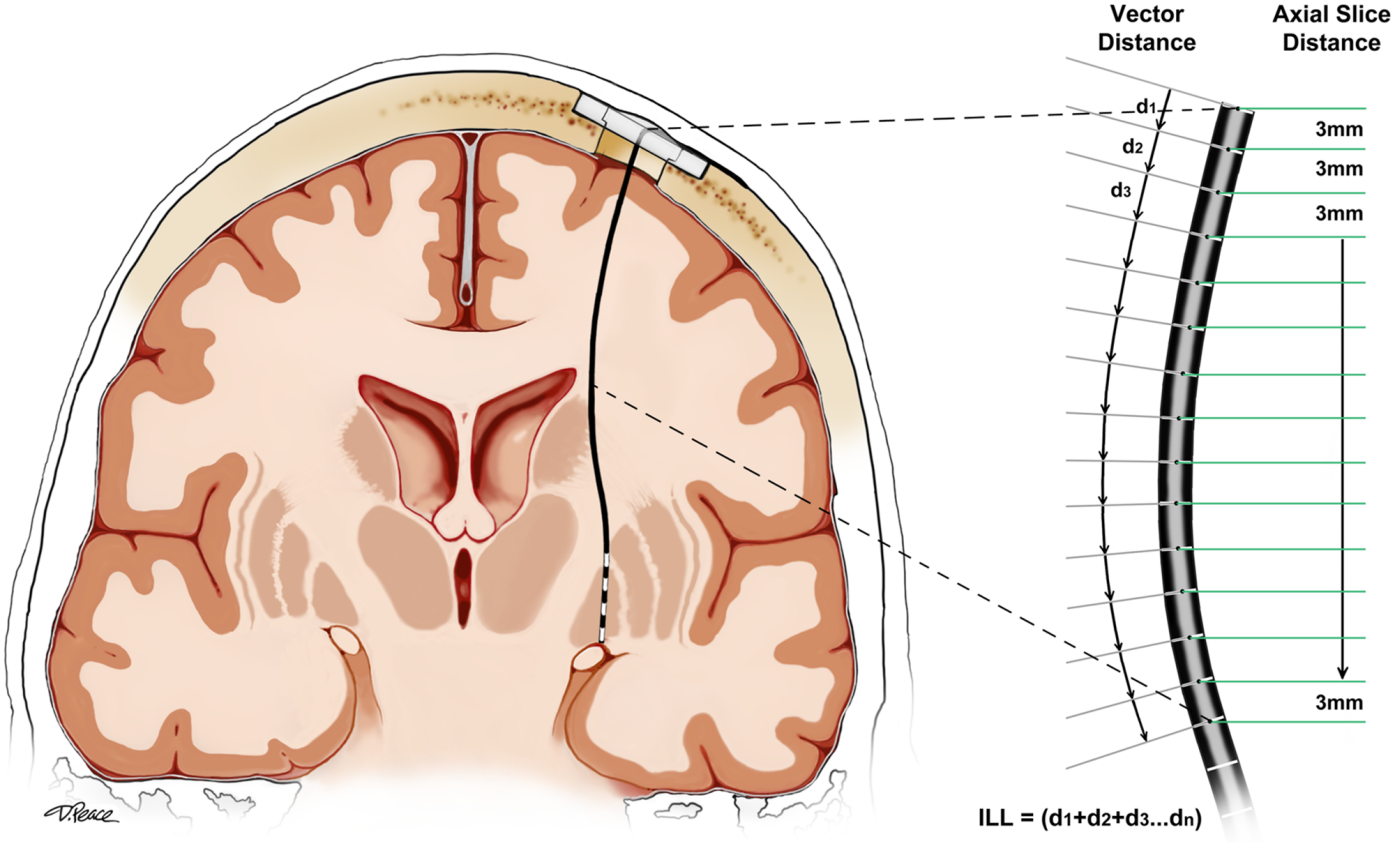
Methodology for measurement of the intracranial length of a curved DBS lead. ILL = intracranial lead length.

The curvilinear intracranial lead length (L) was determined on each lead localization CT by measuring the coordinates of the DBS lead cross-section on axial CT slices at 3 mm intervals from the level of the outer table of the skull to the tip of the DBS lead. The vector distance between each measured point and the next on the DBS lead was calculated and the vector distances were summed to determine the intracranial DBS lead length (Fig 2). The ΔL for a given lead was defined as the difference between the intracranial lead length measured on the initial post-operative lead localization CT and that measured on the delayed lead localization CT for that lead.

The ventricular indices (a surrogate for degree of brain atrophy) were measured using the first preoperative CT scan. An Evans’ index was calculated by dividing the maximal frontal horn width by the maximal inner diameter of the skull in the same plane [25, 26], and the ventricular index was calculated by dividing the maximum cranial width by the maximum frontal horn width as measured in the same plane. The cella-media index was calculated by dividing the minimum width between the lateral ventricles at the cella-media level by maximum external width of the skull [27, 28].

#### Statistical analysis

**Fig 3:**
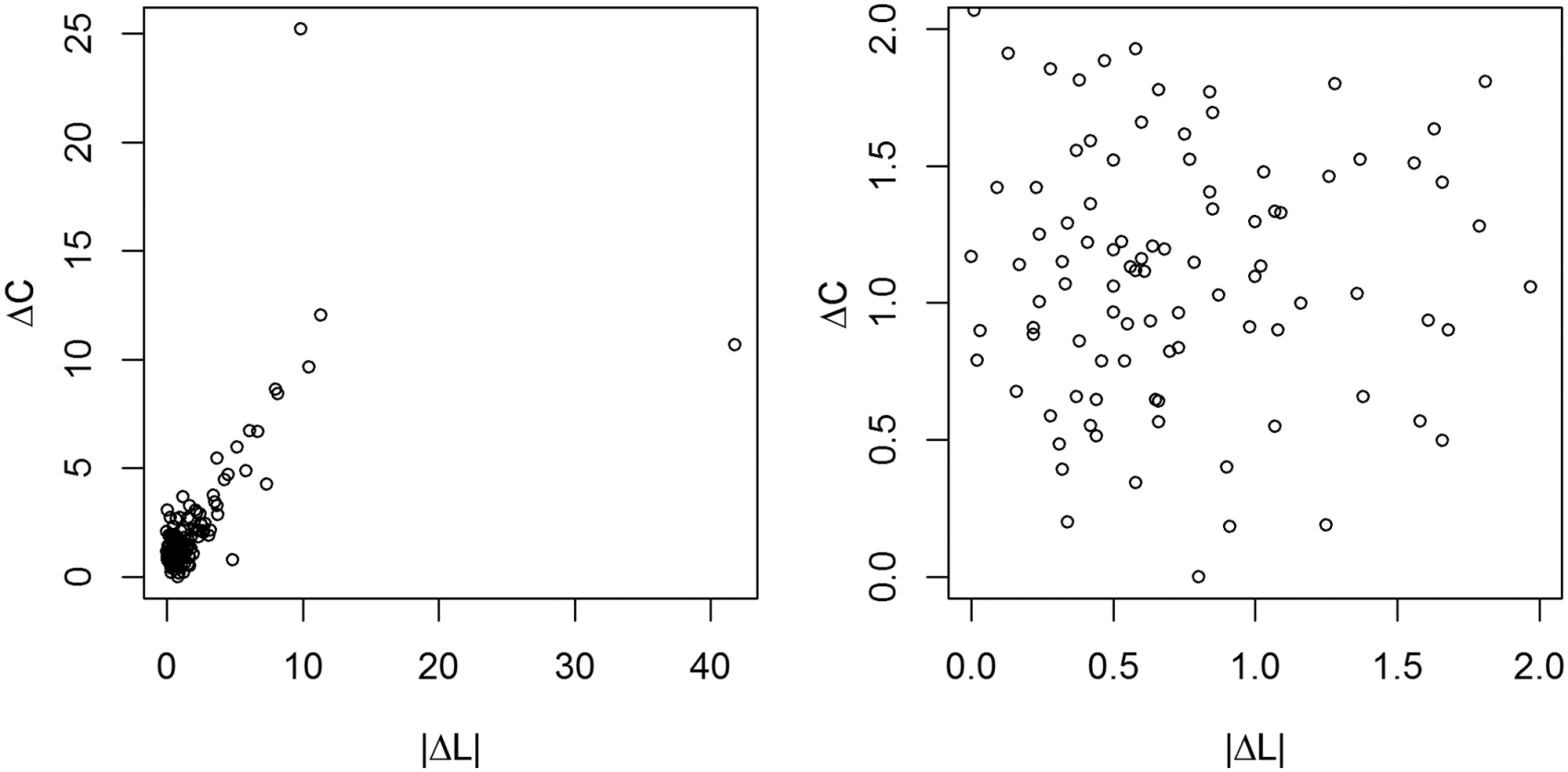
Correlation between ΔC and |ΔL|. A (left). Scatter plot of all values for ΔC and |ΔL|. The overall Spearman (non-parametric) correlation between ΔC and |ΔL| was 0.55 (p < .0001). B. (right) is an enlargement of the area near (0,0)–observations for which both ΔC and |ΔL| are less than 2. The correlation is tiny (0.06) and non-significant (p = 0.562). (ΔC = vector distance between the measured lead tip locations on serial CT scans, |ΔL| = measured change in intracranial lead length on serial CT scans).

We used the R statistical software package (R Foundation for Statistical Computing, Vienna, Austria, V.2.15.0) for statistical analysis in this study. First, we estimated the error in our measurement of ΔC and ΔL to validate our methodology. Since known values of ΔC and ΔL were not available for comparison, we estimated measurement error by identifying a subset of observations for which it was reasonable to believe the probe did not move (i.e., the true value of ΔC and ΔL was 0). We studied the correlation between ΔC and the absolute value of ΔL at various ΔC and |ΔL| cutoff values. Among subjects who experienced electrode movement, we expected to observe a correlation between ΔC and |ΔL|, while among subjects who experienced no probe movement, we would expect the observed ΔC and |ΔL| measurements to reflect random measurement error, resulting in little or no correlation. We identified observed ΔC and |ΔL| cutoff values below which there was a near-0 correlation between the two variables (Fig 3). We assumed that the observed ΔC and |ΔL| values among these patients were observations of pure measurement error, and we used these observations to estimate the error in measuring ΔC and ΔL.

For all comparisons between the migration and no-migration groups, we used Fisher’s exact test to determine whether diagnoses, gender, DBS targets, and DBS sides (left vs right) were associated with migration, and we also used the Mann-Whitney test to determine associations between migration and the following variables: age, days between surgeries, ventricular indices and clinical outcomes.

## Results

### Patient demographics

A database search yielded 135 cases where patients had undergone high resolution CT imaging enabling precise localization of an implanted DBS lead (or leads) at two time points separated by at least three months. Of 141 cases identified in the database in which the patient underwent multiple lead implantation procedures separated by at least three months, 9 cases were excluded, leaving 138 DBS leads implanted in 132 cases available for analysis. The 9 cases were excluded from the study for the following reasons: one pediatric case, four cases of re-implantation into the same target following explantation due to infection, two cases with poor quality CT scans due to motion artifact, and two cases missing post-operative CT scans following surgery. Among the included 132 cases, unilateral DBS leads were measured in 126 patients and bilateral DBS leads were measured in 6 patients, all of whom were dystonia patients.

Among the 132 cases evaluated, 18 were dystonia, 16 were ET, and 104 were PD cases. There were 45 females and 93 males. The DBS targets included the globus pallidus interna (GPi) (39 cases), the subthalamic nucleus (STN) (83 cases), and the ventral intermediate nucleus (Vim) of the thalamus (16 cases). The initial DBS lead was implanted in the left hemisphere in 85 cases, and the right hemisphere in 53 cases. The mean and median ages were 57.9±12.8 and 61, respectively. The interval between the two lead localization CT scans used for the lead migration measurement was 412±306 days (median = 266). The distribution of the cases as they occurred in each year, as well as the results of ventricular size measurement, and other characteristics are summarized in Table 2.

**Table 2:** ΔC, intracranial lead length, and ΔL.

			Intracranial lead lengths		
Diagnosis	Target	ΔC (mm)	1^st^ CT scan	2^nd^ CT scan	ΔL (mm)
**Dystonia**	GPi	4.0±6.2 (2.1)	81.4±3.9 (81.0)	82.5±11.6 (80.0)	-1.0±10.8 (-0.58)
**ET**	VIM	2.1±2.3 (1.4)	83.1±6.2 (81.7)	82.3±5.0 (81.4)	-0.83±3.1 (-0.26)
**PD**	GPi	2.1±2.1 (1.3)	83.6±3.5 (84.1)	82.2±3.8 (82.3)	-1.4±2.6 (-0.34)
	STN	1.7±1.5 (1.4)	87.5±4.7 (88.1)	87.0±5.0 (86.5)	-0.47±2.1 (-0.17)
**Overall**		2.2±2.8 (1.4)	85.6±5.2 (86.1)	85.2±6.5 (85.0)	-0.45±4.4 (-0.28)

These values are mean ± SD (median).

### Estimation of measurement errors

A scatterplot of all data is shown in Fig 3-A. The overall Spearman (non-parametric) correlation between ΔC and |ΔL| was 0.55 (p < .0001). The correlation was clear and strong for large values of ΔC and |ΔL|, but for the majority of points in the clump near the point (0,0), the relationship was ambiguous. Another plot (Fig 3-B) was performed and designed to show an enlargement of the area near (0,0)–observations for which both ΔC and |ΔL| were less than 2. The correlation was very small (0.06) and non-significant (p = 0.562).

The correlation p-values for subsets of the data that include only those observations with both ΔC and |ΔL| values below various cutoff points revealed that correlations did not begin to trend toward significance until ΔC > 1.5. The plot for ΔC cutoff = 1.5 revealed that correlations did not approach significance for any |ΔL| cutoff for the subset of the data. However, there was one observation with |ΔL| > 2 and ΔC < 1.5. This large |ΔL| value (about 5 mm) may have indicated DBS lead displacement, and as a result we set the |ΔL| cutoff at 2 mm to exclude this possibility. Thus, in order to estimate measurement error, we made the assumption that for observations with ΔC < 1.5 and |ΔL| < 2, the true probe displacement was 0.

As a result, estimated measurement errors in ΔC and |ΔL| were 0.92±0.35 mm and 0.72±0.35 mm, respectively. We, therefore, defined significant lead migration as greater than 3 mm changes in both of ΔC and |ΔL|. Furthermore, since each contact on the DBS lead used in this study is 1.5 mm long and the space between contacts is also 1.5 mm, we assumed that a lead migration greater than 3.0 mm might also be expected to significantly affect programming parameters, and ultimately have an impact on clinical outcomes.

### Incidence and risk factors

**Fig 4:**
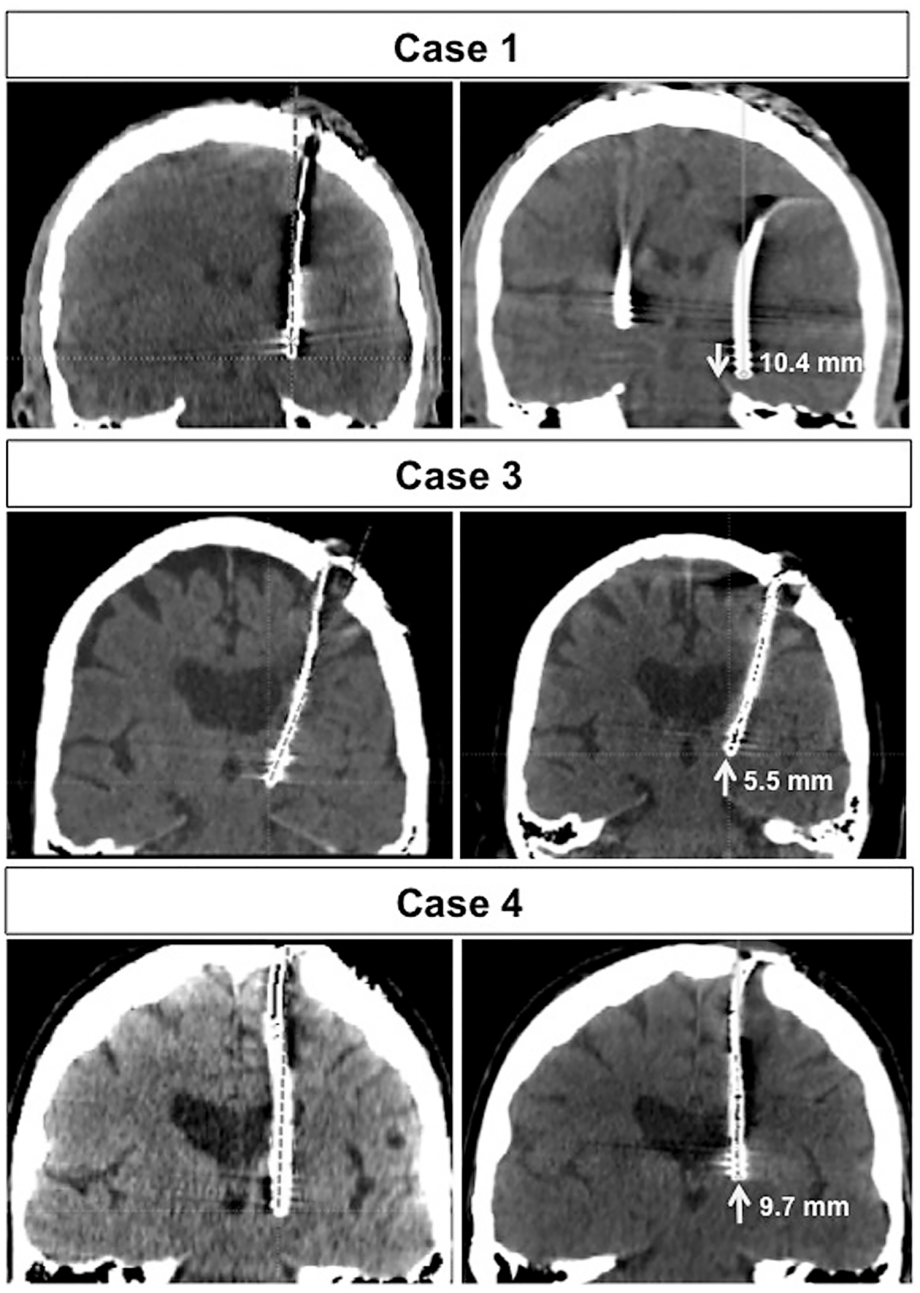
Illustration of an identified DBS lead migration. A: As seen on the initial postoperative scan showing optimal lead position. B: As seen on the targeting CT in preparation for delayed implantation of the contralateral lead. In the majority of cases with significant lead migration, ΔC was nearly equal to ΔL.

The mean ΔC and ΔL were 2.2±2.8 mm (median = 1.4) and 0.45±4.4 mm (median = 0.28) (dorsal), respectively. The details of the ΔC and ΔL values are summarized in Table 3. After careful measurement, 17 leads (12.3%) in 16 patients (12.1%) were determined to have significantly migrated based on these criteria. These 16 cases included two dystonia, two ET, and 12 PD cases. Only one DBS lead migrated ventrally, and the other 16 leads migrated dorsally. As shown in Table 3, ΔC and ΔL were almost identical in most cases of significant lead migration, suggesting that the observed lead migrations in this series resulted from a failure of fixation of the DBS lead at its point of egress from the skull, rather than because of increased curvature of the lead within the head as previously postulated. (Fig 4). According to our prospectively acquired DBS complication database, only four DBS leads were previously known to have migrated after implantation. Although DBS lead migration was discovered in several cases based on the follow-up CT scans, only four of the 17 leads were actually surgically revised. The measurements and reported complications among the 16 cases of DBS lead migration have been summarized in Table 3. Among the statistically analyzed factors, only the year of surgery was found to correlate with incidence of lead migration. Notably, there was an increased incidence of DBS lead migration during the three-year period from 2008 to 2011, with a strikingly high incidence of 50% (6 of 12 cases reviewed) in 2009.

**Table 3:** Characteristics of patients with lead migration.

Case	Diagnosis	Target	Side	Days	ΔC (mm)	ΔL (mm)	Reported Complications
**1**	Secondary dystonia	GPi	L	476	10.7	41.8	Ventral lead migration due to jerky head movement. Second surgery was performed to replace the lead.
**2**	Secondary dystonia	GPi	R	784	12.0	-11.3	Dorsal lead migration on the left. Second surgery was performed to replace the left lead.
		GPi	L	784	25.2	-9.9	
**3**	ET	VIM	L	56	5.5	-3.7	Dorsal lead migration. Second surgery was performed to replace the lead. Twiddler syndrome.
**4**	ET	VIM	L	138	9.7	-10.4	Dorsal lead migration. Second surgery was performed to replace the lead.
**5**	PD	STN	R	126	4.5	-4.2	
**6**	PD	STN	L	266	6.7	-6.7	
**7**	PD	STN	R	526	8.4	-8.2	
**8**	PD	STN	L	259	3.3	-3.7	
**9**	PD	GPi	R	245	6.7	-6.1	
**10**	PD	GPi	L	259	4.6	-7.4	
**11**	PD	STN	R	763	4.9	-5.8	
**12**	PD	STN	L	217	4.7	-4.5	Subdural hematoma with no surgical intervention, superficial infection treated with oral antibiotics
**13**	PD	STN	L	203	6.0	-5.2	Subdural hematoma with no surgical intervention, intraoperative air embolus
**14**	PD	STN	L	721	3.7	-3.4	Superficial infection on the chest incision
**15**	PD	GPi	L	238	8.6	-8.0	Intraoperative seizure
**16**	PD	STN	L	539	3.5	-3.6	Venous infarction

### Clinical outcomes with lead migration

Overall percent improvements with DBS were 6.8±35.5% (median = 6.9) in UDRS scores in the dystonia cohort, 40.0±13.5% (median = 37.9) in TRS scores in the unilateral VIM DBS ET cohort, and 16.4±31.4% (median = 21.2) in UPDRS scores in the unilateral DBS PD cohort. The clinical outcomes (percent improvement) in patients with lead migration were 19.3±19.8% (median = 30.7) in the dystonia cohort and 0.77±36.0% (median = 10.3) in the PD cohort while clinical outcomes in patients without lead migration were 6.6±32.5% (median = 28.0) in the dystonia cohort and 18.6±30.1% (median = 21.8) in the PD cohort. There were only two cases of significant lead migration in the ET cohort, and the complication was detected in the early postoperative period prior to performing the formal TRS evaluation. There was a significant difference in the clinical outcome between dystonia cases with and without lead migration (p = 0.035). Though the difference in clinical outcomes in the PD cohort seemed large, the difference was not statistically significant (0.084). The details are summarized in Table 4.

**Table 4:** Clinical outcomes (%improvement).

Diagnosis	Overall Mean±SD (Median)	Migration Group Mean±SD (Median)	Non-migration Group Mean±SD (Median)	p-value
**Dystonia (UDRS)**	16.8±35.5 (6.9)	19.3±19.8 (30.7)	26.6±32.5 (28)	0.035
**ET (TRS)**	40.0±13.5 (37.9)	NA	40.0±13.5 (37.9)	NA
**PD (UPDRS)**	16.4±31.4 (21.2)	0.77±36.0 (10.3)	18.6±30.1 (21.8)	0.084

NA = not applicable, TRS = tremor rating scale; UDRS = unified dystonia rating scale; UPDRS = unified Parkinson’s disease rating scale

**Fig 5:**
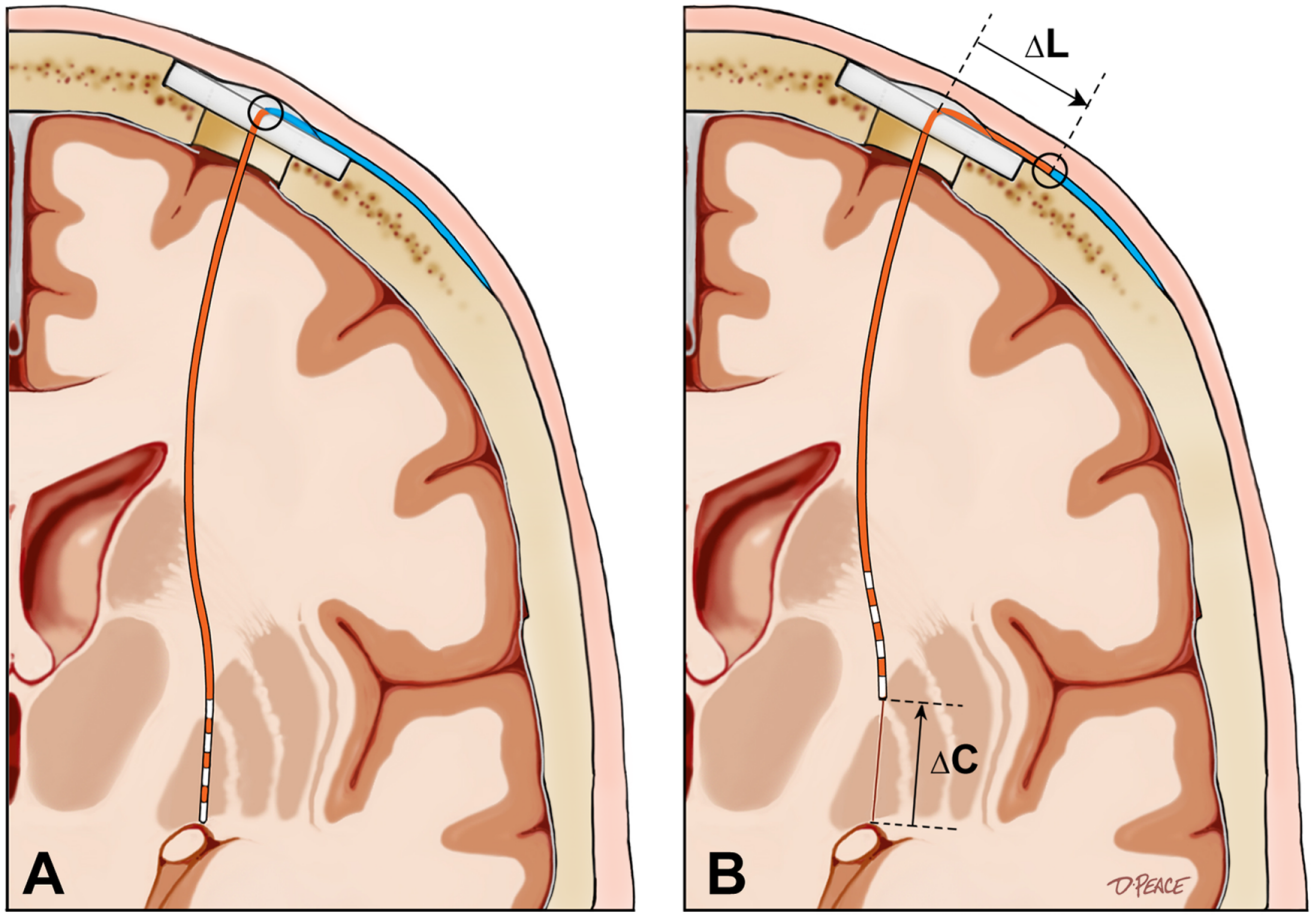
Representative images of dislocated leads. **Cases described in the text. Case 1: A unique ventral lead migration due to repetitive dystonic head movements. Cases 3 and 5: Dorsal lead migrations secondary to “twiddler syndrome”**. Arrows and numbers indicate the direction and the distance of the movement, respectively.

The DBS programming parameters of the patients with lead migration are summarized in Table 5. Observed parameter changes may indicate the time point when the lead migration occurred in some cases. For example, the most ventral contact (contact 0) was used at both of the initial and most recent DBS programming session in case 4, and this may indicate that the migration had occurred prior to initial programming. In this case, the patient did not have optimal tremor suppression even with the high voltage (5.6V). Investigation of this suboptimal DBS outcome included lead localization imaging, which disclosed the significant lead migration. This patient underwent successful re-operation to remove the ineffective lead and re-implant a DBS lead in the optimal location (Fig 5). In most cases of significant lead migration in Table 5, ventral contacts (contact 0 or 1) were used throughout the clinical course, and we assumed the lead migration occurred before the initial programming session.

**Table 5:** DBS programming parameters and threshold levels of patients with lead migration.

Case	Side	Initial Programming	Last Programming
**Dystonia cases**			
**1**	L	C+, 1-, 2V, 180μsec, 60Hz	C+, 1-, 2.8V, 150μsec, 100Hz
**2**	R	C+, 1-, 2.8V, 150μsec, 60Hz	C+, 1-, 3.3V, 180μsec, 60Hz
	L	C+, 1-, 2.8V, 150μsec, 60Hz	C+, 1/2-, 3.5V, 450μsec, 185Hz
**ET cases**			
**3**	L	C+, 3-, 2.8V, 90μsec, 135Hz	2+, 0-, 1.5V, 90μsec, 185Hz
**4**	L	C+, 0-, 2.5V, 90μsec, 185Hz	C+, 0-, 5.6V, 120μsec, 160Hz
**PD cases**			
**5**	R	C+, 2-, 2.4V, 90μsec, 135Hz	C+, 2-, 2.8V, 90μsec, 160Hz
**6**	L	C+, 1-, 2V, 120μsec, 135Hz	3+. 1/2-, 2.6, 120μsec, 160Hz
**7**	R	C+, 2-, 3V, 90μsec, 135Hz	1+, 0-, 3.5V, 90μsec, 185Hz
**8**	L	C+, 2-, 2.5V, 90μsec, 160Hz	C+, 2-, 2.8V, 90μsec, 185Hz
**9**	R	C+, 2-, 3V, 90μsec, 135Hz	3+, 1-, 2.9V, 60μsec, 185Hz
**10**	L	C+, 2-, 2V, 90μsec, 135Hz	C+, 1-, 1.4V, 90μsec, 135Hz
**11**	R	C+, 1-, 2.9V, 90μsec, 135Hz	C+, 2-, 2.9V, 90μsec, 160Hz
**12**	L	C+, 1-, 1.8V, 90μsec, 160Hz	C+, 1-, 2.4V, 90μsec, 160Hz
**13**	L	C+, 1-, 4V, 120μsec, 145Hz	3+, 1-, 3.5V, 120μsec, 160Hz
**14**	L	C+, 0-, 2V, 90μsec, 135Hz	C+, 1-, 2V, 120μsec, 130Hz
**15**	L	C+, 1-, 4V, 120μsec, 145Hz	C+, 1-, 3V, 90μsec, 160Hz
**16**	L	C+, 1-, 2.3V, 90μsec, 135Hz	C+, 1-, 2.7V, 90μsec, 145Hz

The threshold levels were measured with monopolar settings.

The study cohort includes several noteworthy case scenarios involving delayed lead migration. Case 1 (a generalized dystonia patient) initially enjoyed substantial benefit from DBS therapy and the thresholds for stimulation-induced side effects during early programming were felt to be appropriate, but clinical efficacy was lost at a subsequent follow-up visit, and a very unusual ventral lead migration was discovered on a lead localization CT (Fig 5). This patient underwent successful replacement of the ventrally displaced, clinically ineffective DBS lead approximately one year after the initial operation. The lead migration in this case was considered to be related to repetitive forceful retrocollic neck movements. This fascinating case has been described in a previous paper [5]. Another unusual example is case 3 (ET patient) in whom twiddler syndrome (repeatedly flipping over the IPG inside of the chest pocket) [29, 30] resulted in dorsal migration of the DBS lead (Fig 5). This patient enjoyed substantial tremor suppression at the initial programming visit and the thresholds at each lead contact were reasonable, however, twiddler syndrome with a sudden loss of clinical effectiveness emerged. A successful re-operation two months after the first operation resolved the issue.

### Complications

Overall, 43 of 132 patients included in this study had complications. Surgery-related complications included inconsequential intraoperative air embolus (2 cases), intraoperative seizure without post-operative sequellae (2 cases), postoperative seizure without long-term adverse effects (7 cases), and transient post-operative mental status decline (15 cases). Symptomatic and asymptomatic intracranial hemorrhages were reported in five and four cases, respectively. Hardware-related complications included a fractured lead or extension cable (7 cases), and twiddler syndrome (3 cases). Deep and superficial infectious complications were reported in 15 cases and in two cases, respectively. The details of these complications are summarized in Table 6.

**Table 6:** Reported complications in the cohort.

Complication	Number of patients
**Surgery-related**	
Air embolus	2
Intraoperative seizure	2
Postoperative seizure	7
Mental status decline (temporary)	15
Symptomatic intracranial hemorrhage	5
Asymptomatic intracranial hemorrhage	4
**Hardware-related**	
Fractured lead or extension cable	7
Twiddler’s syndrome	3
Deep infection (surgical intervention required)	15
Superficial infection (Oral antibiotics)	2

## Discussion

### Discrepancy between reported migration and measured migration

Lead migration has been variably reported at 0–6.3%/patient and 0–3.2%/lead in the literature (Table 7) [11–20, 31, 32], however, few studies have carefully measured lead migration distance and correlated migration with clinical outcomes. In an effort to accurately measure the intracranial lead length despite lead curvature, we meticulously segmented the DBS leads into consecutive three mm axial sections (Fig 2). Our results revealed that 17 leads (12.3% of leads) in 16 patients (12.1% of cases) out of 138 DBS leads (132 patients) had delayed migrations measuring greater than 3 mm, although only four patients had lead migration identified in our prospectively collected DBS complication database. Clinical improvements in ET DBS manifest immediately after initiating stimulation, and suboptimal clinical outcomes could be detected in the early postoperative stage (97 days on average) [33]. Both of the ET migration cases in our cohort underwent rapid revision with improvement. In dystonia cases it was far more complex to judge benefit, as the effectiveness usually emerges in a more delayed fashion [34]. Our series, not unexpectedly, revealed a delay in detection of suboptimal outcomes in patients treated for dystonia (580 days on average). Finally, the outcomes in PD DBS, similar to dystonia, are typically complex and there are many factors (medication, stimulation, behavioral) that may affect outcome. Our series, revealed an intermediate delay in identification of lead migration in PD cases (363 days on average).

**Table 7:** Incidence of lead migration in the literature.

Authors	Year	Number of Patients/Electrodes	Number of patients with migration (incidence: %/patient)	Number of electrodes with migration (incidence: %/electrode)
Joint et al.	2002	39/79	2 (5%)	2 (2.5%)
Oh, et al.	2002	79/124	4 (5.1%)	4 (3.2%)
Kondziolka et al.	2002	66/NA	1	NA
Lyons et al.	2004	80/155	5 (6.3%)	5 (3.2%)
Yianni et al.	2004	133/240	3 (2.3%)	3 (1.3%)
Constantoyannis, et al.	2005	144/204	0	0
Blomstedt and Hariz	2005	119/161	4 (3.4%)	4 (2.5%)
Voges et al.	2006	180/352	5 (2.8%)	5 (1.4%)
Kenney et al.	2007	319/NA	10 (3.1%)	NA
Boviatsis et al.	2010	106/208	1 (0.94%)	1 (0.5%)
Doshi	2011	153/298	4 (2.5%)	4
Baizabal Carvallo et al.	2012	512/856	9 (1.8%)	10 (1.2%)

NA = not available. Large case volume series with electrode implantations greater than 50 were selected for this table.

### Risk factors and clinical impact

**Fig 6:**
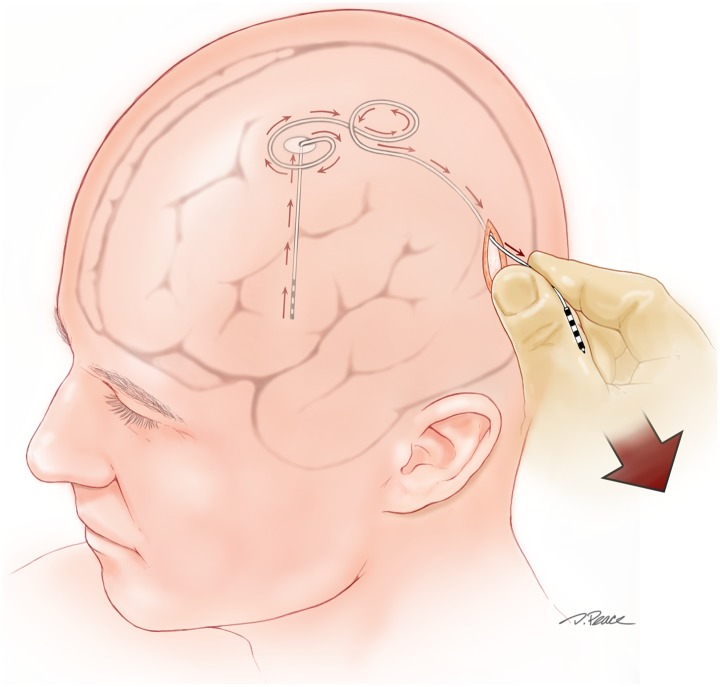
Illustration of a tensile force applied at the DBS connector site resulting in dorsal migration of the intracranial lead. After a few weeks of healing, scalp tethering prevents the subgaleal loops in the lead from uncoiling and results in coaxial transmission of the tension all the way to the StimLoc cap and, if the cap fixation fails, the intracranial lead is displaced.

The hypothesis that dorsal DBS lead migrations result from postoperative curving of the intracranial portion of the lead was not supported by the data. In this series, lead migrations resulted from failure of fixation at the point of egress of the DBS lead from the skull (a StimLoc cap in these cases) when a tensile (or in one case, compressive) force was applied. (Figs 4–6). Most commonly, these lead migrations were iatrogenic, resulting from **an important technical error committed by the surgeon** during delayed implantation and connection of the pulse generator and extension cable to the previously implanted DBS lead. To our surprise and dismay, it has become clear that the majority of the dorsal lead migrations observed in this series were a direct result of the implanting surgeon’s excessive application of tension to the distal aspect of the implanted lead during its connection to the DBS extension cable. (Fig 6) There was a remarkably high incidence of dorsal lead migration in our series between 2008 and 2011. Because the lead materials, the method of cranial fixation, and the timing of staged procedures have remained constant, we attributed the increased frequency of dorsal lead migration over this specific three-year interval to a variation in surgical technique. The implanting surgeon (KDF) acknowledges a change in his technique during this period that was intended to simplify connection of the implanted lead to the tunneled extension cable, whereby more forceful pulling on the end of the DBS lead exposed a longer segment of the distal lead and facilitated placement of the required connection cover and connection of the lead to the extension. This more forceful pulling produced a gradual buildup of tension, occasionally followed by a sudden release and exposure of a few extra centimeters of the distal lead. This release phenomenon was assumed at the time to result from the uncoiling of a distal loop in the subgaleal segment of the lead. Many intra-operative observations since that time have confirmed that this was a misconception. It has become clear that after a few weeks of healing, scalp tethering prevents the subgaleal loops in the lead from uncoiling and instead results in transmission of the applied tension along the axis of the lead. The observed sudden release presumably results when the cumulative friction between the lead and surrounding scalp tissue is overcome, resulting in sudden sliding of the lead along its circuitous subgaleal path, with transmission of the tension all the way to the StimLoc cap. If lead fixation at the cap fails, the intracranial lead is also dorsally displaced. The observation that dorsal lead migration was generally present only on the delayed scan (typically 6 months after IPG implantation) and not on the initial lead localization scan (performed one month after lead implantation and one day prior to the IPG implantation procedure) supports this explanation. The unfortunate lesson learned from this project has, of course, resulted in a corrective modification of our surgical technique. Currently, we make a 4-cm long scalp incision over the proximal aspect of the palpable dummy connector (typically in the region of the parietal boss) and the distal DBS lead to expose enough distal lead that forceful pulling is not necessary to obtain adequate exposure for connection. This longer, more proximal incision also enables us to drill a groove in the skull to accommodate the permanent connector. This technique minimizes the prominence of the implanted connector, improves patient comfort and satisfaction, minimizes the risk of delayed scalp erosion at the connector site, and minimizes the risk of dorsal lead migration or lead fracture from twiddling or repetitive head movements by providing a point of fixation of the connector that prevents a tensile force applied to the extension cable from being transmitted to the implanted DBS lead. An alternative technical solution involves staged implantation of the pulse generator and extension cable prior to intracranial lead placement, such that the lead extension is present at the parietal location, ready to be connected to the newly implanted lead. An improved burr hole cover/lead fixation device with more robust pullout strength could also minimize the incidence of dorsal lead migration.

Our cohort also confirmed two rare, but previously reported causes of lead migration. Yianni et al. reported a higher incidence of lead migration in dystonia patients and stated that severe head movements may have contributed to the etiology [8], as was true in our case one. Twiddler syndrome has also been reported as a risk factor for migration with the repetitive flipping of the IPG in the chest resulting in the transmission of a tensile force to the intracranial lead [30]. This was observed in case three.

As shown in our results, DBS lead migration can result in a significant loss of therapeutic benefit. This was significant in our dystonia cases, and even though the analysis of the PD cohort did not reveal a statistically significant difference between the migration and the non-migration group, there was a clear trend, and there were certainly individual patients who lost DBS effectiveness after lead migration. Clinicians should be aware that DBS lead migration represents an uncommon, but important adverse event that can adversely affect DBS outcomes. There are multiple potential etiologies for lead migrations, but careful surgical technique (as described above) can mitigate this problem. Improved methods of securing the DBS lead, as well as better capping devices, may also decrease the incidence of migration [35, 36].

### Limitations of present study

Because our study lacked known values for comparison, our estimation of measurement error was based on a reasonable, but unverifiable assumption, and could therefore only be considered as a “best guess.” In selecting cutoffs for observed ΔC and |ΔL| that are more than 3 times larger than our estimated errors, we feel confident that the subjects included in our migration group experienced significant electrode movement, while those in the no-migration group experienced little or no movement. Uitti et al. reported measurement errors within 1.4 mm when they compared the location of a thalamotomy lesion on MRI scan between two time points [37], and this study is in general supportive of our criteria. It is however possible that some subjects were misclassified if our methodology underestimated the true measurement error.

There were several other limitations to our study. Our cohort was large but only single-center, and the case selection for surgery could have been biased. Additionally, the incidence of lead migration may not be generalizable. The risk of iatrogenic dorsal lead migration (as described above) will presumably be higher at centers like ours where IPG implantation is delayed for a few weeks after lead implantation. DBS teams that implant the IPG shortly after DBS lead implantation—before the lead has scarred into the subgaleal space—will presumably have a lower risk of surgeon error resulting in dorsal lead migration.

## Conclusions

We have exploited a unique opportunity to evaluate the incidence of delayed lead migration following DBS implantation in a series of 132 patients with 138 DBS leads. In this cohort, 17 DBS leads (12.3% of leads) in 16 patients (12.1% of cases) were found to have migrated postoperatively by more than 3 mm. Excessive tension applied to the lead during its connection to the IPG, repetitive head movements due to dystonia, and twiddler syndrome were risk factors associated with DBS lead migration. Lead migration adversely affected clinical outcomes in several cases. Lead migration may, in the majority of cases, be a preventable complication, and careful surgical technique (as described herein), and possibly an improved lead fixation device, could diminish the incidence of this adverse event. In instances of poor response to DBS therapy, lead localization imaging should be obtained to ensure that the lead is appropriately positioned. Routine lead localization imaging in the early post-operative period, combined with careful evaluation of both clinical effectiveness and thresholds for stimulation induced adverse effects, may be useful in identifying DBS lead migration cases, and in some cases, may lead to earlier surgical correction.
